# Supplementary material for: Adaptation of a Potyvirus Chimera Increases Its Virulence in a Compatible Host through Changes in HCPro
Source: Plants (Basel). 2022 Aug 30;11(17):2262. doi: 10.3390/plants11172262 (PMC9460054; doi:10.3390/plants11172262)
Supplement: Supplementary file 1 [file plants-11-02262-s001.zip › plants-1839334-supplementary.pdf]

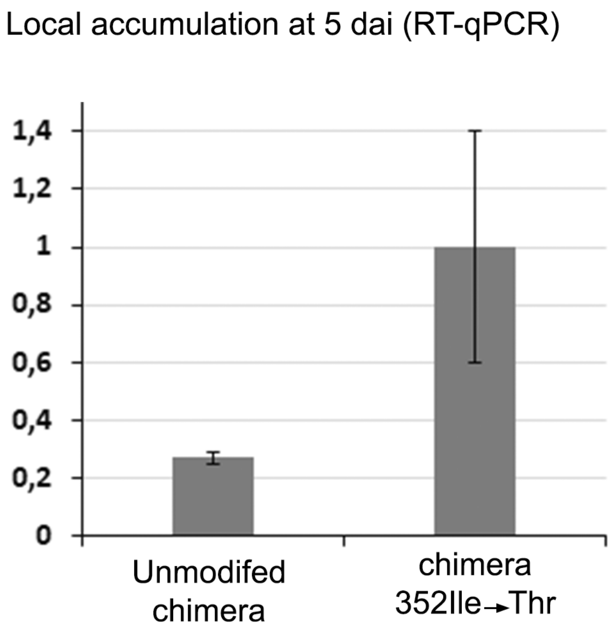

Figure S1. RT-qPCR amplification of viral genomic RNA to assess relative accumulation of the unmodified chimera vs. chimera 352Ile→Thr locally in infiltrated discs (within the same leaf) at 5 dai. The samples analyzed are the same than those shown in Figure 6.

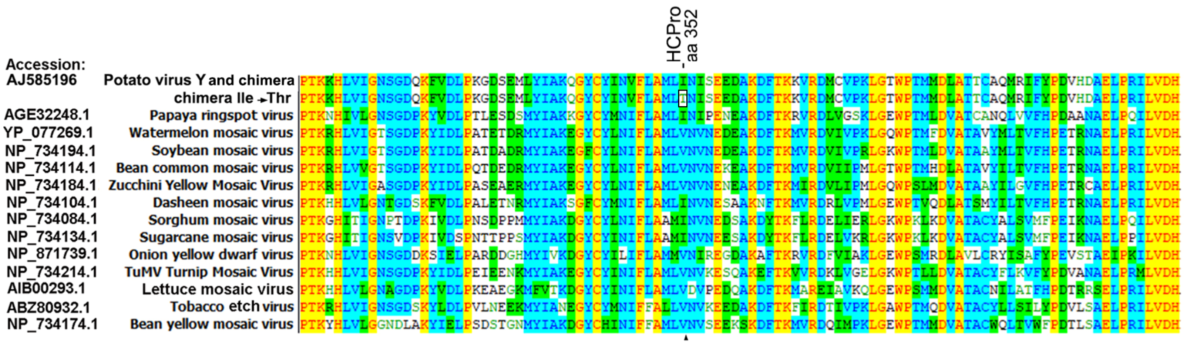

Figure S2. Alignment of HCPro amino acid sequences expressed by several Potyvirus species at PVY HCPro amino acid position 352, and its surrounding region. The non-conservative substitution Ile→Thr found in the chimera infecting plant A and later incorporated into chimera 352Ile→Thr is unique, absent from the potyvirus sequences compared.
